# The Unintended Consequences of Clinical Trials Regulations

**DOI:** 10.1371/journal.pmed.1000131

**Published:** 2009-11-17

**Authors:** Alex D. McMahon, David I. Conway, Tom M. MacDonald, Gordon T. McInnes

**Affiliations:** 1 Dental School, Faculty of Medicine, University of Glasgow, Glasgow, Scotland; 2 Ninewells Hospital & Medical School, University of Dundee, Dundee, Scotland; 3 Gardiner Institute, Faculty of Medicine, University of Glasgow, Glasgow, Scotland

## Abstract

Alex McMahon and colleagues critique the International Conference on Harmonisation (ICH) guidance on good clinical practice (GCP), arguing that it is having a disastrous effect on noncommerical randomized clinical trials in Europe.

**Summary Points**

- Trial regulations are damaging noncommercial research and patients.
- The International Conference on Harmonisation (ICH) version of Good Clinical Practice (GCP) is inapplicable to most noncommercial research.
- ICH GCP is not usually legally binding (as conceded by the regulatory authorities in the UK).
- Other parts of the world should learn a lesson from the misguided trial regulations that have been created in Europe.

## Introduction

The experience of clinical researchers worldwide indicates that a major obstacle to undertaking academic research is the ever-increasing bureaucracy attached to the process. Recent changes in research governance were intended to ensure that clinical trials are safe and informative. However, the regulatory burden is now obstructing high quality science and has become the biggest single threat to research carried out in academia [1]. We illustrate here this international problem by reference to the regulations imposed by the European Union and the incorporation of these restrictions into UK national law concerning Good Clinical Practice (GCP).

GCP sounds like a sensible idea that all researchers would aspire to. However, it used to have a technical meaning in the pharmaceutical industry when attempting to license new pharmacological entities with government agencies such as the Food and Drug Administration in the US. This technical meaning was to follow (specifically) the International Conference on Harmonisation (ICH) document on GCP [2], to facilitate the conduct of multinational drug trials sponsored by the pharmaceutical industry. The harmonisation process was developed over many years by the industry. ICH GCP and the attendant regulations apply to medicinal products for human use only. Nonmedicinal treatments such as psychological interventions and surgery are exempt. There are serious concerns that the onerous procedural requirements for data management and documentation stipulated by ICH are deterring academic research where registration of a new pharmaceutical entity is not an objective.

The rigid bureaucracy of GCP as defined by ICH has already been recognised as an impediment to clinical research, resulting in an effect opposite to that originally envisaged [3]. The ICH guideline on GCP provides extremely detailed instructions on data management and reporting of trials, as would be appropriate for drug companies seeking to license a new pharmaceutical entity with the relevant drug agencies. The true purpose of GCP, based upon foundations in the original Declaration of Helsinki, is to protect patients from unethical research, ensure that patients provide informed consent, and to conduct all trials to the highest possible standard. Few would dispute the need to incorporate the highest standards of GCP in all clinical trials, but does full application of ICH facilitate this goal? Unfortunately the standards of ICH GCP have been rolled out across Europe for all trials of medicinal products in humans in a series of regulations.

## Regulation in Europe

By May 2004, The European Directive 2001/ 20/ EC on clinical trials (“The Directive”) had been adopted across the European Union [4]. Implicit in the title of The Directive is the implementation of GCP and articles of The Directive include informed consent, ethics committees, reporting of adverse events, and national inspection of trials. The Directive was incorporated into the law of the United Kingdom in the Medicines Act [5] and is described on the Medicines and Healthcare Products Regulatory Agency (MHRA) website [6]. “The conditions and principles of GCP which apply to all clinical trials” are “based on” the ICH guideline. The European Directive 2005/28/EC attempts to provide more detailed guidelines on GCP [7]. This GCP Directive instructs that the ICH guideline on GCP should be “taken into account.” The content of this directive appears advisory rather than prescriptive. Whether it was intended for academic clinical trials to be included is uncertain. The Medicines Act stipulates only the general principles section of ICH rather than the more detailed sections [5],[6]. The GCP Directive states that noncommercial research as carried out by public bodies can “make the application of certain of the details of good clinical practice unnecessary or guaranteed by other means,” with member states “providing for specific modalities.” However, the eventual draft guidance mainly discusses treatment labelling and trial documentation [8].

Following the 2005 “GCP Directive,” the UK Medicines Act had to be amended. The first amendment (August 2006) mainly addressed technical matters such as document handling and payment of fees to MHRA but with no mention of “taking into account” the ICH GCP document [9]. The second amendment (December 2006) was specifically designed to enable trials in emergency medicine where informed consent could not be obtained from an incapacitated patient [10]. Explanatory memoranda for both amendments are on the MHRA website [11],[12]. When queried, the MHRA clinical trials helpline (clintrialhelpline@mhra.gsi.gov.uk) made the following statements: “ICH is the standard expected by the CHMP for trials used for centralised licensing submissions”; “ICH is only mentioned in the recital of European Directive 2005/28/EC. The recital is not legally binding”; “Some member states chose to make ICH GCP their legal standard – the UK did not.” Despite this, trial centres in the UK are being aggressively audited to ICP GCP standards.

## Damage to Noncommercial Trials

The academic and public research communities were alarmed at the prospect of the directive of May 2004 [13]–[17]. These regulations were clearly created for the benefit and/or regulation of the pharmaceutical industry [18],[19], and it was inevitable that the number of noncommercial trials would decrease [20]. It was also anticipated that the pharmaceutical industry itself would avoid the extra costs by moving trials out of Europe and into less developed countries [21]. The potential problem for noncommercial research was clearly recognised by the MHRA, which appeared powerless to intervene [22]. A second GCP Directive followed and three UK laws were passed to implement these Directives. The process for ever-increasing bureaucracy appears to be on-going with no sign of conclusion. In the meantime there has been real damage to patient care across the European Union. The EU was warned that the directive would severely impair trials in emergency medicine, because of the difficulty of obtaining a legal representative to give informed consent [23]. The problem with the directive was first recognised in Austria [24], but applies to the whole of Europe and was not resolved in the UK until 2006.

Despite concerns, the amended Medicines Act was duly passed in May 2004. Were the initial concerns misplaced, and once embedded did the new regulations begin to work in the desired manner? The evidence suggests not. By the end of 2005 one group in Cardiff noted that they had “almost stopped doing drug studies” [25], and it was estimated that the number of European trials submitted for grants or ethical review had fallen by 30% to 50% and that the proportion of noncommercial trials was reduced from 40% to 14% [26]. Meanwhile, there does not seem to be much harmonisation of laws across the European Union, one of the main goals of the whole exercise [27],[28]. The ability of European centres to compete with the better funded US noncommercial trials has been damaged, perhaps irreversibly.

The largest independent cancer research network in Europe (EORTC) has reported that the number of new trials dropped from 38 in 2001, to 19 in 2004, to seven in 2005; trial costs have increased by 85% and trial initiation was five months slower [29],[30]. Senior oncologists have concluded that cancer patients in the future “should be worried” [28]. This report attracted letters stating that The Directive had also led to the abandonment of a trial to address fibromyalgia, and of a trial of melatonin, and it was eroding the normally very high rates of recruitment into paediatric cancer trials (an area of very little interest to the pharmaceutical industry) [31]–[33]. There were around ten to 20 studies in paediatric oncology starting per annum before implementation of The Directive, and this has now dropped to a handful [34]. The future of noncommercial paediatric trials in general is in difficulty as the number of studies “will decrease dramatically in the future” [35]. As Mitchell notes: “For children with cancer the effect of this directive has been appalling” [36].

There was some hope that a European Regulation that was designed to help promote paediatric trials would help the noncommercial sector [35]. This directive, EU Regulation 1901/2006 “on medical products for paediatric use” came into force in January 2007 and is clearly focussed on the pharmaceutical industry's ability to patent and market new treatments for children [37],[38]. In any case the Paediatric Regulation insists on full compliance with the Clinical Trial Directive! There is some doubt as to the utility of this Regulation for even industrial trials [39].

A survey of eight cancer clinical trial centres in the UK also found that the cost of noncommercial trials had doubled, trials have been delayed, and staff were demoralised in many trial centres [40]. Since funds are often collected directly from the public by charity appeals, public money is being spent. It always difficult to fund clinical trials in this way, with some of the real costs being absorbed or cross-subsidised in department accounts. Considerably more money is now required, and only projects that are attractive to the pharmaceutical industry are likely to proceed. Since outcomes are often better in patients taking part in clinical trials, countless thousands receiving care outside of trials are therefore having their health damaged because of the reduced recruitment into trials. This is particularly true of areas that are of no interest to the pharmaceutical industry.

## Discussion

Drug trials initiated in academia have similarities with conventional pharmaceutical company trials but also important differences. The primary aims of academic trials are to improve patient care rather than to develop new pharmacological entities. Surely, these objectives are of equivalent or of greater importance to society? In the conduct of both types of trial, GCP is important but the need for intrusive bureaucracy to ensure harmonisation is much less relevant to academic studies usually carried out at a single site. In the past, the pharmaceutical industry might have sponsored such research but The Directive makes this much less likely. The requirements of The Directive have dissuaded Universities from taking on this role. Accumulating evidence suggests that many research units and individual researchers have withdrawn from noncommercial randomised clinical trials altogether because of The Directive.

ICH standards are expected by pharmaceutical companies for licensing submissions. According to the MHRA (by personal communication) the legally binding parts of the trial regulations include the following: staff must be qualified, procedures should be in place to ensure quality; data should be accurate and verified; patient confidentiality should be maintained; patient consent is documented. This short list is just common sense in doing a professional job of doing good quality trials. There is no mention of the massive list of SOPs or the entire training and auditing industry that has sprung up around the ICH documents. We should not forget that the GCP Directive itself conceded that that the conditions of noncommercial trials render the “application of certain of the details of good clinical practice unnecessary or guaranteed by other means.”

There appears to be some room for manoeuvre in the legislation with regard to the interpretation of “good clinical practice.” Instead of accepting each new layer of bureaucracy the academic community should clarify these developments, using legal advice where necessary. The cost-effectiveness of the type of procedures that ICH GCP generates is untested. For example, it has been estimated that the cost of a single “data query” is US$150. Robert Califf notes that this is a “colossal waste of money,” and that a more effective and scientific method would be to use sampling and the statistical process control techniques that are found in other industries (such as engineering and manufacturing) [41]. In short, there is no evidence that the intense bureaucracy of centralised politically driven procedures for ICH GCP has improved the care of trial participants in any way.

As bureaucracy increases, the efficiency of the trial process decreases. There is no evidence base to support the implementation of the burden of this extra bureaucracy, and academics are perplexed as to the utility of this bureaucracy. There is a phrase for overinterpretation of regulatory advice that is sometime used when discussing EU competition and tendering rules called “regulatory creep.” Hearn and Sullivan have pointed out that “regulatory creep” is being caused by overinterpretation of trial “guidance” [40]. Instead of regulatory creep in clinical trials, we would like to see some “regulatory retreat” where academics try to ensure that the interpretation of any rules and procedures that are not mandated by law are the most favourable for academic research whilst ensuring patient safety. Researchers funded with public money should seek to adhere to the legal minimum required to carry out research rather than the bureaucratic maximum as suggested by ICH GCP. Ideally there would be a completely different and alternative version of GCP for noncommercial trials, especially those that study medications that have been on the market for decades and have treated millions of patients.

Of course these problems apply directly to all trials that plan to include a European centre. Other parts of the world now have a new advantage for attracting clinical research, at the expense of European patients. A further advantage is that other parts of the world can use Europe as a “test bed” to demonstrate the dreadful problems of misguided regulation, and can choose to avoid these difficulties if they wish. The dangers of applying ICH GCP to trials where it should be considered to be inapplicable have been demonstrated in this paper. A first step would be for noncommercial researchers to recognise that ICH GCP is not usually legally binding in a particular country (e.g., the UK). We would favour a combined tactic of lobbying to simplify and “regulatory retreat” and perhaps we could then look forward to more “specific modality” exceptions for noncommercial trials in future legislation.

## Abbreviations

GCP: good clinical practice
ICH: International Conference on Harmonisation
MHRA: Medicines and Healthcare Products Regulatory Agency
